# Effects of irbesartan on phenotypic alterations in monocytes and the inflammatory status of hypertensive patients with left ventricular hypertrophy

**DOI:** 10.1186/s12872-021-02004-7

**Published:** 2021-04-20

**Authors:** Jingsi Zhang, Lina Yang, Yanchun Ding

**Affiliations:** 1 Department of Cardiology II, The Second Hospital of Dalian Medical University, Dalian, 116023 China; 2 Department of Cardiology, Central Hospital of Huludao, Huludao, China

**Keywords:** Hypertension, Left ventricular hypertrophy, Monocyte phenotype, Inflammation, Irbesartan

## Abstract

**Background:**

Circulating monocytes and tissue macrophages play complex roles in the pathogenesis of hypertension and the resulting target organ damage. In this study, we observed alterations in the monocyte phenotype and inflammatory state of hypertensive patients with left ventricular hypertrophy (LVH) and studied the effects of irbesartan in these patients. This study might reveal a novel mechanism by which irbesartan alleviates LVH, and it could provide new targets for the prevention and treatment of hypertensive target organ damage.

**Methods:**

CD163 and CD206 expression on monocytes and IL-10 and TNF-α levels in the serum of hypertensive patients with or without LVH and of healthy volunteers were detected. Furthermore, we treated monocytes from the LVH group with different concentrations of irbesartan, and then, CD163, CD206, IL-10 and TNF-α expression was detected.

**Results:**

We found, for the first time, that the expression of CD163, CD206 and IL-10 in the LVH group was lower than that in the non-LVH group and healthy control group, but the TNF-α level in the LVH group was significantly higher. Irbesartan upregulated the expression of CD163 and CD206 in hypertensive patients with LVH in a concentration-dependent manner. Irbesartan also increased the expression of IL-10 and inhibited the expression of TNF-α in monocyte culture supernatants in a concentration-dependent manner.

**Conclusions:**

Our data suggest that inflammation was activated in hypertensive patients with LVH and that the monocyte phenotype was mainly proinflammatory. The expression of proinflammatory factors increased while the expression of anti-inflammatory factors decreased. Irbesartan could alter the monocyte phenotype and inflammatory status in hypertensive patients with LVH. This previously unknown mechanism may explain how irbesartan alleviates LVH.

*Trail registration* The study protocols were approved by the Ethical Committee of the Second Affiliated Hospital of Dalian Medical University. Each patient signed the informed consent form.

## Background

Hypertension is one of the most common chronic diseases, and the number of patients with hypertension is increasing year by year. According to a report on cardiovascular disease in China 2016, there were approximately 270 million patients with hypertension in China, accounting for approximately 1/5 of the global number of patients with hypertension, and 30% of the patients with hypertension were complicated by left ventricular hypertrophy (LVH) [1]. LVH is considered to be an important marker of target organ damage in hypertension and is an important predictor of coronary heart disease, heart failure and stroke. Patients with LVH have a significantly increased risk of cognitive impairment, atherosclerosis, atrial fibrillation and other diseases. LVH is currently used as an independent risk factor for the assessment of the occurrence and prognosis of cardiovascular diseases [2]. As a result, the prevention and alleviation of LVH has become a long-term goal of hypertension treatment.

The occurrence of LVH is related to various mechanisms. Multiple factors are involved in the formation of LVH, including age, sex, obesity, heredity, hemodynamics, oxidative stress, endothelial function, neurohormonal factors, and inflammatory immunity [3]. The relationship between the inflammatory response and LVH has attracted increasing attention in recent years.

The immune-inflammatory response is activated throughout cardiovascular diseases and is related to the development of hypertensive target organ damage [4, 5]. The monocyte/macrophage system is the main effector of innate immunity, and it plays an important role in mediating hypertensive target organ damage through blood pressure-independent mechanisms. When the inflammatory response is activated, reactive oxidants and many cytokines, such as tumor necrosis factor-α (TNF-α), interleukin-1β (IL-1β), interleukin-6 (IL-6), and monocyte chemoattractant protein-1 (MCP-1), are produced. These cytokines then promote the aggregation and activation of monocyte/macrophage cells and other inflammatory cells in the target organs of hypertension and further promote the inflammatory response as well as the progression of hypertensive target organ damage.

Irbesartan is an angiotensin II (Ang II) receptor blocker that can specifically antagonize the angiotensin type 1 receptor (AT1R). A meta-analysis suggested that angiotensin receptor antagonists (ARBs) could significantly alleviate LVH, and the left ventricular mass index (LVMI) is decreased on average by 3.2%. Moreover, the effect of ARBs was better than that of other antihypertensive drugs, including angiotensin-converting enzyme inhibitors (ACEIs), calcium antagonists, diuretics and β-blockers [6].

In this study, we observed phenotypic alterations in monocytes and the expression of inflammatory cytokines in hypertensive patients with LVH to explore the relationship between the inflammatory immune response and hypertensive LVH. We further observed the effect of irbesartan on the changes in the peripheral blood monocyte phenotype and inflammatory status in hypertensive patients with LVH, which might be a previously unknown mechanism whereby irbesartan causes LVH regression.

## Methods

### Subjects and grouping

A total of 59 patients with primary hypertension admitted to our department from December 2016 to December 2017 were included. According to the 2010 China Hypertension Guideline, high blood pressure (BP) was defined as a systolic blood pressure (SBP) higher than or equal to 140 mmHg and/or diastolic blood pressure (DBP) higher than or equal to 90 mmHg without antihypertensive drugs. Hypertensive subjects who already received antihypertensive drugs were included regardless of their BP values. The duration of their hypertension was not considered. The main exclusion criteria were the presence of clinical or laboratory evidence of congestive heart failure, atrial fibrillation, hyperthyroidism, renal insufficiency, sleep apnea–hypopnea syndrome, previous stroke, significant cardiac valve disease, previous myocardial infarction, history of coronary bypass, neoplastic disease or secondary hypertension. In addition, patients who took ACEIs or ARBs within the past 2 weeks were not included.

According to the 2016 Asia Expert Consensus on Diagnosis and Treatment of Hypertension complicated with Left Ventricular Hypertrophy, patients with LVMI > 115 g/m^2^ for men and LVMI > 95 g/m^2^ for women were considered to have LVH. Among the patients with hypertension, there were 30 patients with LVH (21 males and 9 females) and 29 patients without LVH (13 males and 16 females). In the same period, 30 healthy volunteers were admitted, including 16 males and 14 females.

The study protocols were approved by the Ethical Committee of the Second Affiliated Hospital of Dalian Medical University. The project was carried out on the premise of protecting the rights and interests of the subjects according to the Declaration of Helsinki. All subjects were fully informed and signed informed consent forms. Participants were divided into three groups: the LVH group included hypertensive patients with LVH, the non-LVH group included hypertensive patients without LVH, and the normotensive control group included healthy volunteers.

### Study design

The patient characteristics, including sex, age, height and body weight, SBP, and DBP of all of participants were recorded, and body mass index (BMI) was calculated on the basis of weight and height (kg/m^2^). Total cholesterol (TC), triglyceride (TG), high-density lipoprotein cholesterol (HDL-C), low-density lipoprotein cholesterol (LDL-C), homocysteine (Hcy), high-sensitivity c-reactive protein (hs-CRP), and β-2 microglobulin (β-2MG) were measured.

Two-dimensional guided M-mode recording was performed through the parasternal window according to the guidelines of the American Society of Echocardiography. The following parameters on the M-mode echocardiogram were evaluated: left ventricular end diastolic diameter (LVEDd, mm), interventricular septal diastolic thickness (IVSTd, mm), and left ventricular posterior wall diastolic thickness (LVPWTd, mm). Left ventricular mass (LV mass) was calculated according to Devereux’s adjusted formula: LVM (g) = 0.8 × 1.04 × [(LVEDd + LVPWTd + IVSTd)^3 ^− LVEDd^3^] + 0.6. Left ventricular mass index (LVMI) (g/m^2^) = LVM (g)/body surface area (BSA) (m^2^). BSA was calculated according to the following formulas: BSA for men = 0.0057 × height (m) + 0.0121 × body weight (kg) + 0.0882 and BSA for women = 0.0073 × height (m) + 0.0127 × body weight (kg) − 0.2106. The monocytes in the peripheral blood were isolated by the Ficoll-Hypaque density separation method (Sigma, Germany). The expression of CD163 and CD206 in the monocytes was detected by flow cytometry (BD, USA). The concentrations of IL-10 and TNF-α in the serum and supernatant of the monocyte cultures were detected by ELISA (Abcam, UK).

### Statistical analyses

SPSS 22.0 software was used for the statistical analysis. All continuous variables were tested for a normal distribution. The data that were normally distributed are presented as the mean ± standard deviation. Comparisons between groups for normally distributed data were performed with Student’s *t* test. Analysis of variance (ANOVA) was used for comparisons among multiple groups, and the LSD method was used for pairwise comparisons. The data that were not normally distributed were tested using a nonparametric test and are presented as medians. Counting data were analyzed by chi-square tests. Values of *P* < 0.05 were considered to be statistically significant.

## Results

### Patients’ characteristics

There were no significant differences in age, sex, BMI, TC, LDL-C, HDL-C, or TG among the three groups (*P* > 0.05). The SBP and DBP of the LVH and non-LVH groups were higher than those of the control group (*P* < 0.05), but there was no significant difference between the LVH and non-LVH groups (*P* > 0.05). Hcy and β-2MG in the LVH group were higher than those in the control group (*P* < 0.05). The LVM (male), LVM (female), LVMI, and serum hs-CRP of the LVH group were higher than those of the non-LVH group and control group, and those of the non-LVH group were higher than those of the control group (*P* < 0.05) (Table 1).

**Table 1:** Patients’ characteristics

	Control (n = 30)	Non-LVH (n = 29)	LVH (n = 30)	F	*P*
Gender (male/female)	16/14	13/16	21/9		
Age (years)	58.53 ± 10.17	59.07 ± 11.79	58.03 ± 14.93	0.28	0.755
LVM (male)	133.02 ± 30.97	145.19 ± 25.43*	219.45 ± 29.23*^#^	30.84	< 0.001
LVM (female)	105.40 ± 16.28	131.62 ± 23.18*	209.29 ± 27.38*^#^	37.13	< 0.001
LVMI	74.01 ± 11.87	74.12 ± 13.02	115.95 ± 9.98*^#^	85.59	< 0.001
SBP	123.75 ± 11.08	146.46 ± 27.63*	155.57 ± 11.65*	4.55	0.015
DBP	73.50 ± 5.06	85.42 ± 9.42*	90.46 ± 10.23*	6.09	0.004
BMI (kg/m^2^)	23.13 ± 1.23	24.62 ± 2.21	24.51 ± 4.06	0.23	0.801
TC (mmol/L)	4.17 ± 0.79	4.69 ± 0.91	4.79 ± 1.03	0.89	0.416
LDL-C (mmol/L)	1.29 ± 0.19	1.39 ± 0.34	1.47 ± 0.54	1.24	0.296
HDL-C (mmol/L)	2.48 ± 0.31	2.83 ± 0.76	2.55 ± 0.74	1.21	0.304
TG (mmol/L)	1.79 ± 1.43	1.89 ± 1.24	1.84 ± 1.01	0.01	0.987
Hs-CRP (mg/L)	11.50 ± 1.29	12.42 ± 3.09*	14.12 ± 4.32*^#^	2.01	0.143
Hcy (umol/L)	1.43 ± 0.65	1.37 ± 0.98	2.87 ± 1.87*	7.82	0.001
β-2MG (mg/L)	1.85 ± 0.47	1.98 ± 0.53	2.70 ± 0.72*	10.69	< 0.001

*P < 0.05 versus control, ^#^*P* < 0.05 versus non-LVH group

### Comparison of CD163 and CD206 expression

**Fig. 1:**
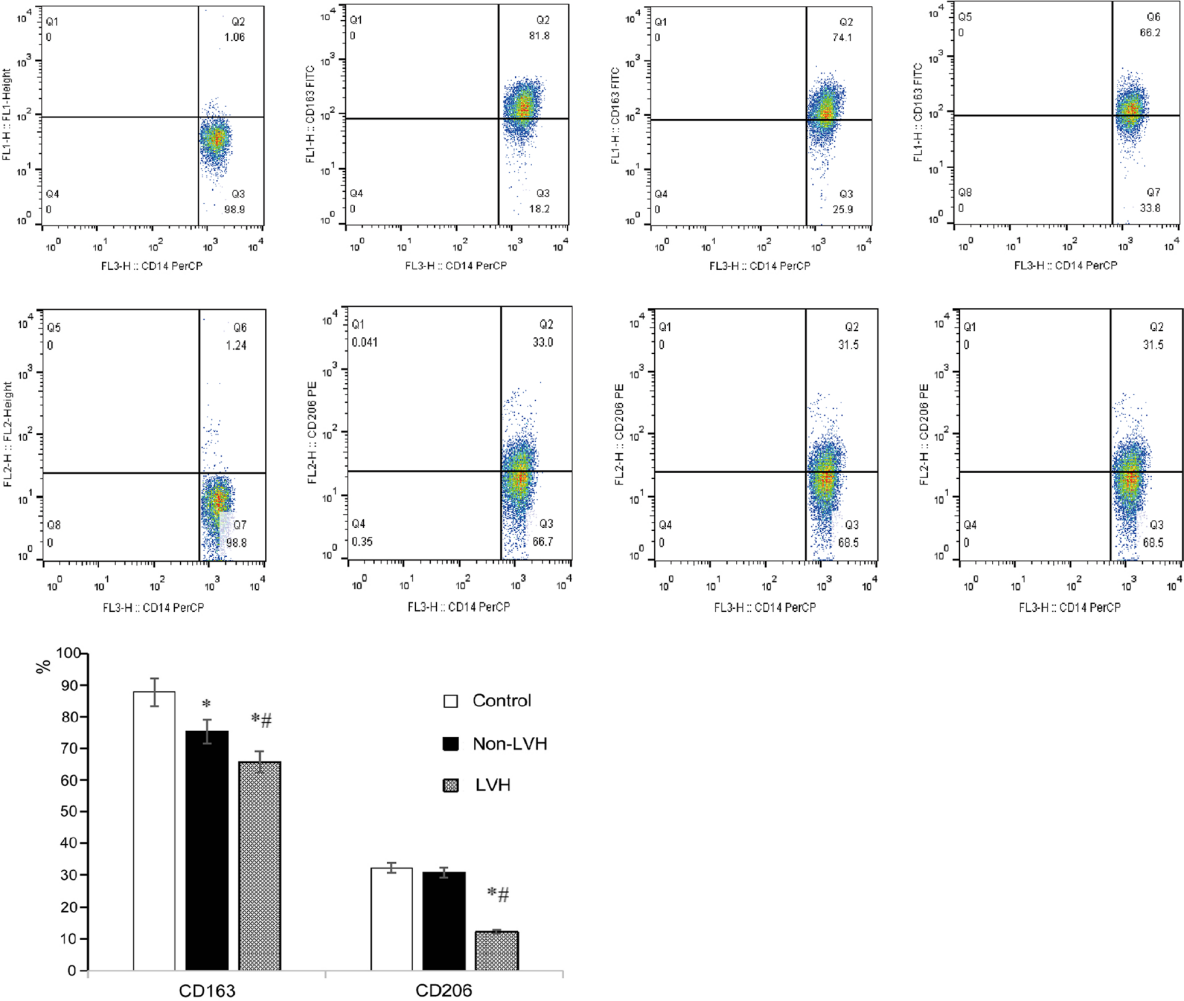
Expression of CD163 and CD206 in monocytes detected by flow cytometry. CD163 and CD206 expression on monocytes of the LVH group was lower than that on monocytes of the non-LVH and control groups (*P* < 0.05). CD163 in the non-LVH group was lower than that in the control group (*P* < 0.05). *P < 0.05 versus control, ^#^*P* < 0.05 versus non-LVH

The expression of CD163 and CD206 on monocytes of the LVH group was lower than that of the non-LVH group and control group (*P* < 0.05). The expression of CD163 in the non-LVH group was lower than that in the control group (*P* < 0.05) (Table 2, Fig. 1).

**Table 2:** Expression of CD163 and CD206

	Control (n = 30)	Non-LVH (n = 29)	LVH (n = 30)	F	*P*
CD163 (%)	87.75 ± 25.32	75.29 ± 18.30*	65.73 ± 17.89*^#^	13.18	< 0.001
CD206 (%)	32.32 ± 7.82	30.81 ± 8.29	12.19 ± 6.07*^#^	8.45	0.002

*P < 0.05 versus control, ^#^*P* < 0.05 versus non-LVH group

### Comparison of IL-10 and TNF-α

**Fig. 2:**
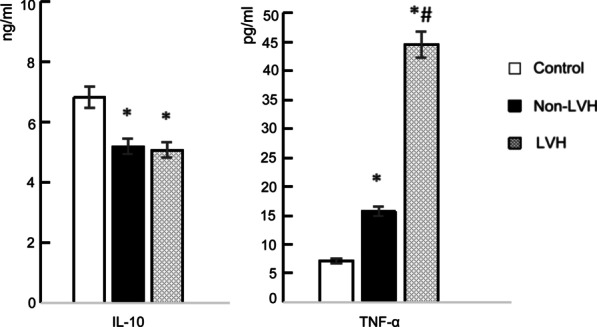
Levels of IL-10 and TNF-α. IL-10 levels in the LVH and non-LVH groups were lower than those in the control group (*P* < 0.05). TNF-α in the LVH group was significantly higher than in the non-LVH and control groups, and the non-LVH group was higher than the control (*P* < 0.05). *P < 0.05 versus control, ^#^*P* < 0.05 versus non-LVH

The concentration of TNF-α in the serum of the LVH group was significantly higher than that of the non-LVH group and control group, and that of the non-LVH group was higher than that of the control group (*P* < 0.05). The concentration of IL-10 in the serum of the LVH group and non-LVH group was lower than that of the control group (*P* < 0.05), but there was no significant difference between the LVH group and the non-LVH group (Table 3, Fig. 2).

**Table 3:** Levels of IL-10 and TNF-α

	Controls (n = 30)	Non-LVH (n = 29)	LVH (n = 30)	F	*P*
IL-10 (ng/mL)	6.82 ± 1.24	5.19 ± 1.05*	5.07 ± 0.85*	4.62	0.019
TNF-α (pg/mL)	7.11 ± 1.61	15.67 ± 7.62*	44.63 ± 17.96*^#^	21.78	< 0.001

*P < 0.05 versus control, ^#^*P* < 0.05 versus non-LVH group

### Effects of irbesartan on monocyte phenotype and inflammatory status in hypertensive patients with LVH

**Fig. 3:**
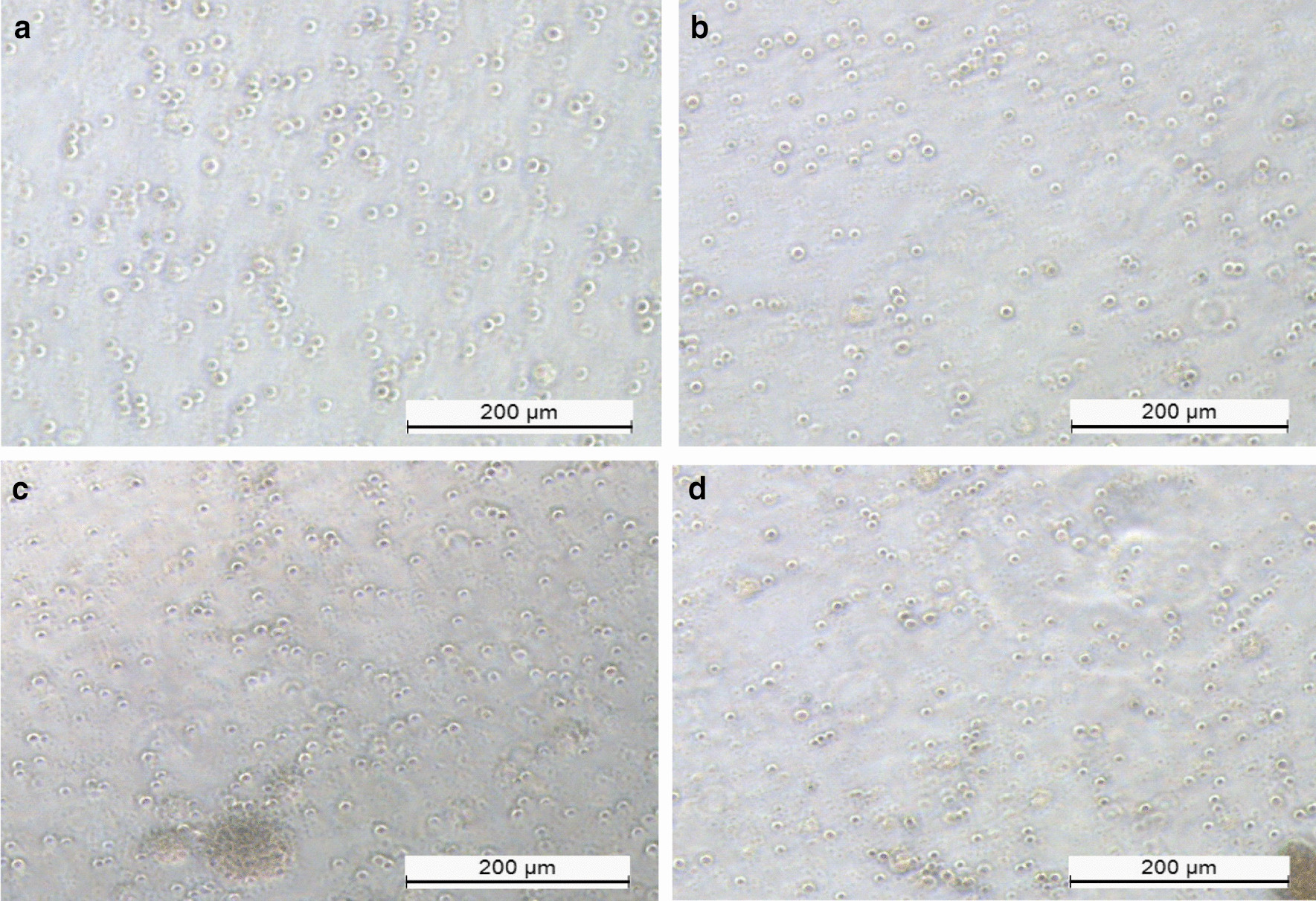
Morphology of monocytes stimulated by different concentrations of irbesartan. **a** Control (blank). **b**–**d** Irbesartan stimulation groups (10^−6^, 10^−7^, 10^−8^ mol/L, respectively)

**Fig. 4:**
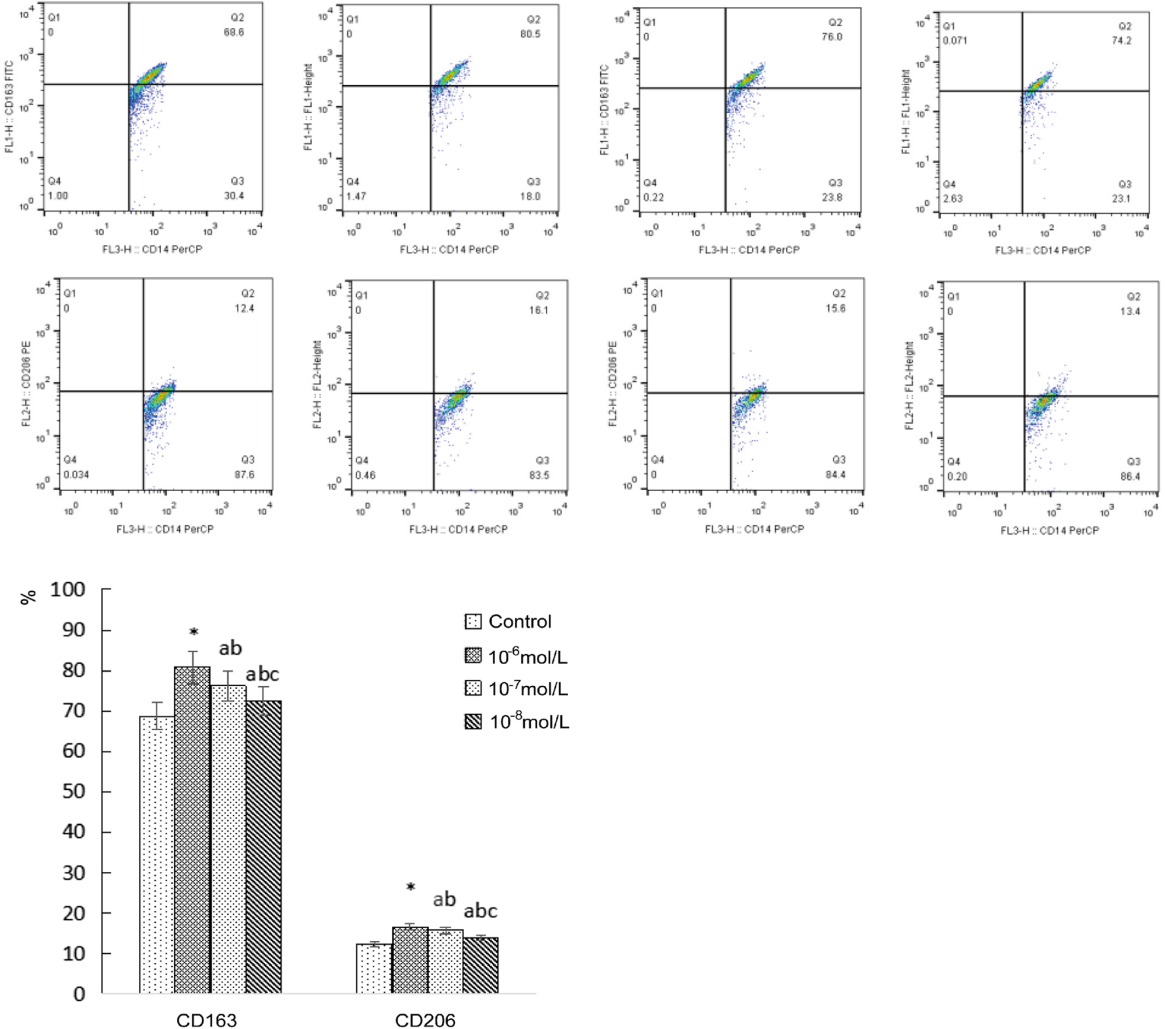
The expression of CD163 and CD206 in LVH patients affected by different concentrations of irbesartan. Irbesartan upregulated the expression of CD163 and CD206 in a concentration-dependent manner, of which the 10^−6^ mol/L group was the most significant (*P* < 0.01). *P < 0.01 versus control, a *P* < 0.05 versus control, b *P* < 0.05 versus 10^−6^ mol/L group, c *P* < 0.05 versus 10^−7^ mol/L group

**Fig. 5:**
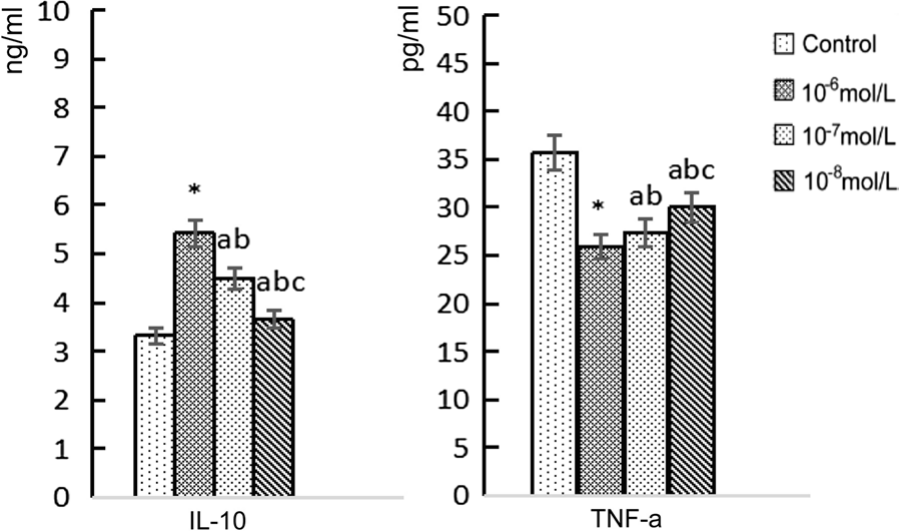
The expression of IL-10 and TNF-α in LVH patients affected by different concentrations of irbesartan. Irbesartan upregulated the expression of IL-10 and inhibited the expression of TNF-α in a concentration-dependent manner, of which the 10^−6^ mol/L group was the most significant (*P* < 0.01). *P < 0.01 versus control, a *P* < 0.05 versus control, b *P* < 0.05 versus 10^−6^ mol/L group, c *P* < 0.05 versus 10^−7^ mol/L group

We stimulated monocytes isolated from the peripheral venous blood of hypertensive patients with LVH for 24 h with irbesartan (10^–6^ mol/L, 10^–7^ mol/L, 10^–8^ mol/L). Then, we observed the morphology of the monocytes (Fig. 3). We found that irbesartan could upregulate the expression of CD163 and CD206 in monocytes of hypertensive patients with LVH in a concentration-dependent manner, of which the 10^–6^ mol/L group was the most significant (*P* < 0.01) (Fig. 4). Irbesartan also increased the expression of IL-10 and inhibited the expression of TNF-α in the culture supernatants of monocyte from hypertensive patients with LVH in a concentration-dependent manner; of these groups, the 10^−6^ mol/L group exhibited the most significant differences (*P* < 0.01) (Table 4, Fig. 5).

**Table 4:** Effect of irbesartan on the expression of CD163, CD206, IL-10 and TNF-α in LVH patients

	Control	Irbesartan			F	*P*
		10^−6^ mol/L	10^−7^ mol/L	10^−8^ mol/L		
CD163 (%)	68.69 ± 14.90	80.76 ± 18.61*	76.19 ± 10.25^ab^	72.45 ± 8.27^abc^	10.74	< 0.001
CD206 (%)	12.42 ± 7.58	16.61 ± 8.69*	15.76 ± 6.83^ab^	13.94 ± 5.17^abc^	12.90	0.028
IL-10 (ng/mL)	3.32 ± 0.90	5.42 ± 0.77*	4.51 ± 0.43^ab^	3.64 ± 0.49^abc^	11.63	< 0.001
TNF-α (pg/mL)	35.69 ± 4.79	25.93 ± 4.44*	27.37 ± 3.19^ab^	30.04 ± 6.21^abc^	4.81	0.011

*P < 0.01 versus control

^a^*P* < 0.05 versus control

^b^*P* < 0.05 versus 10^−6^ mol/L group

^c^*P* < 0.05 versus 10^−7^ mol/L group

## Discussion

LVH is one of the most common target organs damaged by hypertension. It is a compensatory response of the heart to an increased after-load, and is a risk factor for complications and prognosis deterioration. Currently, LVH is used as an independent risk factor for the evaluation of cardiovascular diseases. LVH can lead to a decreased blood flow reserve in coronary arteries and further myocardial ischemia. LVH also reduces left ventricular function, and the cardiac contractile function of LVH patients is lower than that of normal people. A reduction in the energy supply for the hypertrophic cardiomyocytes or other metabolic disorders can aggravate the necrosis and fibrosis of cardiomyocytes. Then, cardiac contraction function and cardiac compliance decrease, leading to the decompensation of cardiac function and the onset of heart failure [7]. LVH could also lead to alterations of cardiac cells and interstitial structures, causing electrophysiological abnormalities. In addition, the imbalance between the coronary blood supply and myocardial area resulting from LVH could lead to cardiac ischemia and changes in the regulation of cardiac autonomic nerve function, which also leads to abnormal myocardial electrical activity and eventually arrhythmia [8]. Previous studies indicated that patients with hypertension who experienced LVH regression reduced their overall cardiovascular event risk by 46%. Prevention and/or alleviation of LVH can reduce the incidence and mortality of cardiovascular diseases and improve the prognosis of patients with hypertension [9, 10].

Hypertension is considered a chronic inflammatory disease. The inflammatory response plays an important role in the occurrence and development of target organ damage in hypertension [11]. The monocyte/macrophage system is the main effector of innate immunity. It can switch back and forth between two states according to the activation mode and immune function. Interferon-γ (IFN-γ) induces M1 macrophage polarization (proinflammatory), which is called the “classical activation type”. Interleukin-4 (IL-4) induces macrophage M2 polarization (anti-inflammatory), which is classified as an “alternative activation type” [12]. The identification of macrophage subsets is based on the expression of specific extracellular and intracellular proteins. The markers of M1 macrophages include CD68, major histocompatibility complex-II (MHC-II), CD80, CD86, CC chemokine receptors, MCP-1, etc. on the cell surface. The markers of M2 macrophages include CD163, CX3C chemokine receptor 1 (CX3CR1), CD206, vascular endothelial growth factor (VEGF) and so on. TNF-α, IL-1β, IL-6, IL-12, IL-18, IL-23 and other proinflammatory factors are highly expressed on M1-type macrophages, while CD163, CD206, IL-10 and other anti-inflammatory factors are highly expressed on M2-type macrophages. Macrophages participate in the progression of hypertension and target organ damage. There are more proinflammatory monocytes in the circulation of patients with hypertension than in those with normal blood pressure. Meanwhile, the levels of some proinflammatory cytokines are increased in patients with hypertension [13–15]. A previous study found that the expression of the M1-type macrophage marker CD68 was markedly elevated in SHRs, while the expression of CD163 and CD206 in SHRs was markedly reduced. After the activation of M2-type macrophages, the M1/M2 ratio was reduced together with a reduction in chemokines such as macrophage inflammation protein 1 (MIP-1α) and MCP-1, and the blood pressure of the SHRs gradually returned to normal. These results strongly suggested that the blood pressure of the SHRs was associated with the phenotype of macrophages and the overall inflammation status [16]. It was previously shown that the level of TNF-α is positively correlated with the degree of LVH in patients with hypertension [17, 18]. A previous study confirmed that inhibition of nuclear factor-κB (NF-κB) could lead to attenuation of LVH both in vitro and in vivo. An in vivo study offered compelling evidence that inhibition of NF-κB activation contributes to the regression of LVH in 2 animal models of hypertension, including spontaneously hypertensive rats and normotensive Wistar Kyoto rats exposed to chronic administration of phenylephrine [19]. Inhibiting the NF-κB pathway could lead to an increase in IL-10, which represents M2-type macrophages. IL-10 may inhibit angiotensin via the NF-κB pathway, thereby inhibiting fibroblast proliferation and collagen synthesis and eventually inhibiting myocardial interstitial fibrosis, indicating that IL-10 may be able to prevent and alleviate hypertension LVH [20]. In this study, the expression of CD163 and CD206 in peripheral blood monocytes and the concentrations of IL-10 and TNF-α in the serum of patients with hypertension and healthy participants were detected. The expression of CD163 and CD206 and the concentration of IL-10 in the LVH group were lower than those in the healthy and non-LVH groups, while the concentration of TNF-α was significantly higher in the LVH group than in the other two groups. These results indicated that inflammation was activated in hypertensive patients with LVH. The expression of pro-inflammatory factors increased and the expression of anti-inflammatory factors decreased, while the monocytes in the blood were mainly pro-inflammatory M1-type in patients with hypertension with LVH. This result suggested that the occurrence and development of LVH were related to alterations in the monocyte/macrophage phenotype and inflammatory status.

Irbesartan is a commonly used antihypertensive drug. Recent studies have shown that oral administration of irbesartan can attenuate hypertension LVH and reduce LVMI in addition to controlling blood pressure [21, 22]. Possible mechanisms include irbesartan inhibiting the renin–angiotensin–aldosterone system (RAAS), reducing the level of AngII and blocking the binding of AngII to AT1R to improve the metabolism of myocardial fibroblasts and reduce myocardial hypertrophy. In addition, irbesartan can improve autonomic nerve function, maintain the balance of sympathetic and parasympathetic nerves, and reduce target organ damage caused by nerve dysfunction. A previous experiment proved that ARB could block the NF-κB signaling pathway and inhibit the release of inflammatory cytokines to prevent or ameliorate target organ damage in hypertension [23]. In this study, monocytes isolated from the peripheral blood of hypertensive patients with LVH were stimulated with different concentrations of irbesartan. A previous study showed that the maximum concentration of irbesartan was 4.43 × 10^–6^ mol/L in healthy subjects receiving 150 mg irbesartan once daily. The time to reach the maximum concentration was 1.5 h, and the elimination half-life was 16 h. In our study, a methyl tetrazolium (MTT) assay was used to determine the drug concentrations, and irbesartan at concentrations of 10^–6^ mol/L, 10^–7^ mol/L, and 10^–8^ mol/L was applied to the monocytes. The concentrations we used in the cell experiment were in the range of plasma concentrations in the human body and could resemble physiological conditions to a considerable extent [24]. In vivo studies should be carried out to verify the results in the future. The expression of CD163 and CD 206 in monocytes and the concentrations of IL-10 and TNF-α in the supernatant of the cells were determined after 24 h of irbesartan treatment. We found that irbesartan could upregulate the expression of CD163 and CD206 in monocytes of hypertensive patients with LVH in a concentration-dependent manner, of which the 10^–6^ mol/L group was the most significant. Irbesartan also increased the expression of IL-10 and inhibited the expression of TNF-α in the culture supernatants of monocytes from hypertensive patients with LVH in a concentration-dependent manner; of these groups, the 10^–6^ mol/L group exhibited the most significant changes. These results indicated that irbesartan could change the monocyte phenotype and inflammatory status in hypertensive patients with LVH. The possible mechanisms include the antioxidant effect of irbesartan. The physicochemical stimulation of monocytes/macrophages by irbesartan at different concentrations leads to autophagy of monocytes/macrophages, and then the functional status of the monocytes/macrophages is changed. Finally, the inflammatory factors released from monocytes/macrophages are also changed [25]. A previous study focused on the role of autophagy in the development of cardiac injury induced by AngII. Autophagy participates in regulating NF-κB activity in macrophages, indicating that improving autophagy might be a novel target for ameliorating hypertensive organ damage [26]. The specific mechanism remains to be further explored. In this study, through in vitro experiments, it was verified for the first time that irbesartan could change the monocyte phenotype and inflammatory status of hypertensive patients with LVH. Meanwhile, irbesartan could control blood pressure and might attenuate LVH by regulating immune inflammation, providing a new direction for hypertensive target organ damage protection. However, there are several limitations of this study, such as the sample size being relatively small, which would easily lead to statistical bias and experimental deviation. Further clinical study is necessary to confirm the results of our in vitro studies.

## Conclusion

In conclusion, inflammation was activated in hypertensive patients with LVH, and the monocyte phenotype was mainly proinflammatory. The expression of proinflammatory factors in the serum increased while the expression of anti-inflammatory factors decreased. Irbesartan could alter the monocyte phenotype and inflammatory status in hypertensive patients with LVH, which may be a previously unknown mechanism by which irbesartan attenuates LVH.

## Data Availability

All relevant data are available from the corresponding author on reasonable request.

## Abbreviations

LVH: Left ventricular hypertrophy
TNF-α: Tumor necrosis factor-α
IL-1β: Interleukin-1β
IL-6: Interleukin-6
MCP-1: Monocyte chemoattractant protein-1
Ang II: Angiotensin II
AT1R: Angiotensin type 1 receptor
ACEIs: Angiotensin-converting enzyme inhibitors
ARBs: Angiotensin receptor antagonists
LVMI: Left ventricular mass index
BP: Blood pressure
SBP: Systolic blood pressure
DBP: Diastolic blood pressure
BMI: Body mass index
TC: Total cholesterol
TG: Triglyceride
HDL-C: High density lipoprotein cholesterol
LDL-C: Low density lipoprotein cholesterol
Hcy: Homocysteine
hs-CRP: High sensitivity creative protein
β-2MG: β-2 Microglobulin
LVEDd: Left ventricular end diastolic diameter
IVSTd: Interventricular septal diastolic thickness
LVPWTd: Left ventricular posterior wall diastolic thickness
LV mass: Left ventricular mass
LVMI: Left ventricular mass index
BSA: Body surface area
ANOVA: Analysis of variance
IFN-γ: Interferon-γ
IL-4: Interleukin-4
MHC-II: Major histocompatibility complex-II
CX3CR1: CX3C chemokine receptor 1
VEGF: Vascular endothelial growth factor
NF-κB: Nuclear factor-κB
RAAS: Renin-angiotensin-aldosterone system
MIP-1α: Macrophage inflammation protein 1
MTT: Methyl tetrazolium
